# Matrix-assisted laser desorption/ionization time-of-flight mass spectrometry for the identification of *Burkholderia pseudomallei* from Asia and Australia and differentiation between *Burkholderia* species

**DOI:** 10.1371/journal.pone.0175294

**Published:** 2017-04-06

**Authors:** Vichaya Suttisunhakul, Apinya Pumpuang, Peeraya Ekchariyawat, Vanaporn Wuthiekanun, Mindy G. Elrod, Paul Turner, Bart J. Currie, Rattanaphone Phetsouvanh, David A. B. Dance, Direk Limmathurotsakul, Sharon J. Peacock, Narisara Chantratita

**Affiliations:** 1 Department of Microbiology and Immunology, Faculty of Tropical Medicine, Mahidol University, Bangkok, Thailand; 2 Department of Clinical Pathology, Faculty of Medicine, Navamindradhiraj University, Bangkok, Thailand; 3 Mahidol-Oxford Tropical Medicine Research Unit, Faculty of Tropical Medicine, Mahidol University, Bangkok, Thailand; 4 Bacterial Special Pathogens Branch, Division of High-Consequence Pathogens and Pathology, National Center for Zoonotic and Emerging Infectious Diseases, Centers for Disease Control and Prevention (CDC), Atlanta, Georgia, United States of America; 5 Cambodia-Oxford Medical Research Unit, Angkor Hospital for Children, Siem Reap, Cambodia; Centre for Tropical Medicine and Global Health, Nuffield Department of Medicine, University of Oxford, Oxford, United Kingdom; 6 Department of Infectious Diseases, Royal Darwin Hospital, Darwin, Northern Territory, Australia; Global and Tropical Health Division, Menzies School of Health Research, Darwin, Northern Territory, Australia; 7 Lao-Oxford-Mahosot Hospital-Wellcome Trust Research Unit, Microbiology Laboratory, Mahosot Hospital, Vientiane, Lao People's Democratic Republic; Centre for Tropical Medicine and Global Health, University of Oxford, Oxford, United Kingdom; 8 London School of Hygiene and Tropical Medicine, London, United Kingdom; 9 Department of Tropical Hygiene, Faculty of Tropical Medicine, Mahidol University, Bangkok, Thailand; Universita degli Studi di Parma, ITALY

## Abstract

Matrix-assisted laser desorption/ionization time-of-flight mass spectrometry (MALDI-TOF MS) is increasingly used for rapid bacterial identification. Studies of *Burkholderia pseudomallei* identification have involved small isolate numbers drawn from a restricted geographic region. There is a need to expand the reference database and evaluate *B*. *pseudomallei* from a wider geographic distribution that more fully captures the extensive genetic diversity of this species. Here, we describe the evaluation of over 650 isolates. Main spectral profiles (MSP) for 26 isolates of *B*. *pseudomallei* (N = 5) and other *Burkholderia* species (N = 21) were added to the Biotyper database. MALDI-TOF MS was then performed on 581 *B*. *pseudomallei*, 19 *B*. *mallei*, 6 *B*. *thailandensis* and 23 isolates representing a range of other bacterial species. *B*. *pseudomallei* originated from northeast and east Thailand (N = 524), Laos (N = 12), Cambodia (N = 14), Hong Kong (N = 4) and Australia (N = 27). All 581 *B*. *pseudomallei* were correctly identified, with 100% sensitivity and specificity. Accurate identification required a minimum inoculum of 5 x 10^7^ CFU/ml, and identification could be performed on spiked blood cultures after 24 hours of incubation. Comparison between a dendrogram constructed from MALDI-TOF MS main spectrum profiles and a phylogenetic tree based on *recA* gene sequencing demonstrated that MALDI-TOF MS distinguished between *B*. *pseudomallei* and *B*. *mallei*, while the *recA* tree did not. MALDI-TOF MS is an accurate method for the identification of *B*. *pseudomallei*, and discriminates between this and other related *Burkholderia* species.

## Introduction

The genus *Burkholderia* is composed of Gram-negative species that are predominantly non-pathogenic environmental saprophytes. A clinically important exception is *Burkholderia pseudomallei*, which causes an estimated 165,000 cases of melioidosis per year worldwide, of which 89,000 are predicted to be fatal [1]. A high proportion of reported cases occur in northeast Thailand, where melioidosis is the second most common cause of community-acquired bacteremia and the third most common cause of death from infectious diseases [2]. *B*. *pseudomallei* receives additional attention because of its biothreat potential [3], and because of infection in travelers returning from endemic regions [4, 5]. In Thailand, *B*. *pseudomallei* is widely distributed in soil and environmental water [6–8] and may be found together with the closely related *B*. *thailandensis*, which is very rarely associated with human disease [9].

Making an accurate diagnosis of melioidosis is key to patient outcome since empiric antimicrobial therapy for sepsis does not include the first-line drugs recommended for melioidosis (ceftazidime or a carbapenem). Diagnosis relies on bacterial isolation and identification since clinical manifestations lack specificity. *B*. *pseudomallei* may be isolated from a range of clinical specimen types, but half of melioidosis cases have positive blood cultures. Direct identification of *B*. *pseudomallei* in positive blood cultures can reduce the time to diagnostic confirmation by 24 hours. This can be achieved using a rapid immunofluorescent assay (IFA), in which the most common antibody described is a monoclonal that recognizes *B*. *pseudomallei* capsular polysaccharide (CPS) [10]. This reagent is also used in a latex agglutination assay that can identify *B*. *pseudomallei* directly in blood cultures, other sample types, and colonies picked from culture media [11]. A monoclonal antibody against CPS has also been incorporated into a lateral flow assay [12], which can detect *B*. *pseudomallei* directly in a range of clinical samples and cultures.

The recent recognition of a *B*. *thailandensis* variant that expresses *B*. *pseudomallei*-like capsular polysaccharide (Bp-like CPS) [13] has important implications for the accuracy of *B*. *pseudomallei* identification assays based on antibody detection of CPS, since this gives a false positive result. The potential relevance of this has increased with the recent finding that *B*. *thailandensis* expressing Bp-like CPS is widely distributed in the environment in Thailand (V. Hantrakul, personal communication). Occasional cross-reacting isolates of *B*. *cepacia* have also been observed (D. Dance, personal communication). Methods that distinguish between *B*. *pseudomallei* and other *Burkholderia* species such as *B*. *thailandensis* include arabinose assimilation, more extended biochemical testing such as that incorporated in the commercial API 20NE biochemical kit (bioMérieux), real-time PCR, and sequencing [14–20]. Automated identification systems are available in many laboratories, although these may misidentify *B*. *pseudomallei* as *B*. *cepacia* [21–24].

Matrix-assisted laser desorption/ionization time-of-flight mass spectrometry (MALDI-TOF MS) has been increasingly introduced into diagnostic laboratories for rapid bacterial identification. The first published study of its application to *Burkholderia* spp. evaluated 10 *B*. *pseudomallei* and 17 *B*. *mallei* isolates and generated reference spectra data [25]. Several case reports have since reported its use to diagnose melioidosis, including infection in returning travelers [5, 26–29]. It has also been reported to differentiate between *B*. *pseudomallei* wild-type and single gene mutants [30], to delineate clustering in a collection of 11 *B*. *pseudomallei* from northeast Thailand [31], and to determine ceftazidime resistance simultaneously with *B*. *pseudomallei* identification [32]. An inter-laboratory trial for the identification of highly pathogenic bacteria using MALDI-TOF MS included one isolate each of *B*. *pseudomallei* and *B*. *mallei* and concluded that a compilation of complete and comprehensive databases with spectra from a broad strain collection was of paramount importance for accurate microbial identification [33]. The largest evaluation of *B*. *pseudomallei* to date was undertaken using 66 *B*. *pseudomallei*. Nearly all of these (62/66 isolates) were initially misidentified as *B*. *thailandensis* using an existing database (DB5627), but an enhanced database subsequently identified all correctly [34]. A limitation of this study was that most of the isolates (63/66) were from a single country (Taiwan), the remaining 3 originating from Beijing, China.

At the time that this study was performed, representation of the 30 *Burkholderia* species in the biotyper database was limited and did not include *B*. *pseudomallei*, *B*. *ubonensis*, *B*. *oklahomensis*, *B*. *humptydooensis* or *B*. *mallei*. This indicates a need to expand the reference database and potentially increase the sensitivity and specificity of the assay. Furthermore, the need remains to undertake an evaluation of MALDI-TOF MS to identify *B*. *pseudomallei* drawn from a wider geographic distribution, which is important because of the genetic distinctiveness of isolates from Asia compared with Australasia and the highly plastic and variable genome, both of which could impact on the performance of MALDI-TOF MS. Previous studies have also lacked the inclusion of *B*. *thailandensis* expressing Bp-like CPS. The aim of this study was to expand the MALDI-TOF MS database with *Burkholderia* spp. and then evaluate this technology using a large collection of *B*. *pseudomallei* together with other *Burkholderia* spp. including *B*. *thailandensis* expressing Bp-like CPS. We added value to our findings by testing its performance using blood cultures spiked with *B*. *pseudomallei*, and evaluating the similarity between *B*. *pseudomallei* clusters arising from MALDI-TOF MS data compared with phylogenetic characterization based on *recA* sequence data.

## Materials and methods

### Bacterial isolates

*B*. *pseudomallei* and other *Burkholde*ria species used in this study are detailed in Table 1. In brief, these included 5 laboratory *B*. *pseudomallei* strains, 581 *B*. *pseudomallei* isolated from the environment, humans or animals, 46 isolates belonging to eight other *Burkholderia* species, and 23 isolates representing a range of other bacterial pathogens. *B*. *pseudomallei* were cultured from humans (N = 550) or other animals (N = 14) with melioidosis, or from the environment (N = 17), and originated from northeast and east Thailand (N = 524), Laos (N = 12), Cambodia (N = 14), Hong Kong (N = 4) and Australia (N = 27). A single isolate was used from each patient, animal or environmental source. The other bacterial species were: *Acinetobacter baumannii*, *Enterobacter aerogenes* NCTC 10006, *E*. *cloacae*, *Enterococcus faecalis* ATCC 29212, *Escherichia coli*, *Haemophilus influenzae* NCTC 11931, *Hafnia alvei*, *Klebsiella oxytoca*, *K*. *pneumoniae* ATCC 700603, *Morganella morganii*, *Neisseria gonorrhoeae*, *Proteus mirabilis*, *Pseudomonas aeruginosa*, *P*. *putida*, *P*. *stutzeri*, *Salmonella enterica* serovar Paratyphi A, *S*. *enterica* serovar Paratyphi B NCTC 3176, *S*. *enterica* serovar Typhi NCTC 8385, *Seratia marcescens*, *Staphylococcus aureus* ATCC 25923, *Stenotrophomonas maltophilia*, *Streptococcus pneumoniae* ATCC 49619 and *S*. *pyogenes*. The original identification of *B*. *pseudomallei* was performed by the submitting laboratories using a range of different methods that reflected variation in clinical and research practice and the lack of a single gold standard method. In Asia, the predominant method used was a combination of colony morphology, antibiotic susceptibility pattern, arabinose assimilation and latex agglutination [35]. Elsewhere, colony appearance followed by API20NE (bioMérieux) was commonly used. Other *Burkholderia* species were identified using one or more of biochemical methods, including *16S rRNA* sequence, *recA* sequence and DNA-DNA hybridization [14–20]. Additional, non-*Burkholderia* species were identified using standard laboratory methodology supplied by the Department of Medical Science, Ministry of Public Health, Thailand. All isolates were shipped in accordance with international guidelines to an accredited BSL-3 laboratory in Bangkok where the evaluation was conducted. Unless otherwise specified, bacteria were cultured on Columbia agar at 37°C in air for 24 h. All isolates were stored in trypticase soy broth (TSB) with 15% glycerol at -80°C.

**Table 1:** *B*. *pseudomallei* and other *Burkholderia* species used in this study.

Bacterial isolates (Total number)	Source or strains				Year of isolation
	Location	Human	Animal	Environment	
*B*. *pseudomallei* (N = 586)	Reference strains (K96243, NR9921, 1106a, 1026b, 576a)	5			Varied
	Sunpasitthiprasong Hospital, Ubon Ratchathani, northeast Thailand	402			2012–2013
	Ubon Ratchathani, northeast Thailand	27		14	1990–2001
	Udon Thani Hospital, northeast Thailand	35			2015–2016
	Khon Kaen Hospital, Khon Kaen, northeast Thailand	19			2015–2016
	Nakhon Phanom Hospital, northeast Thailand	19			2015–2016
	Buddhasothorn Hospital, Chachoengsao, east Thailand	8			2014
	Australia	14	10	3	2002
	Cambodia	14			2006–2008
	Lao PDR	12			2003–2005
	Hong Kong		4		1987 and 2007
*B*. *mallei* (N = 21)	Mongolia (Mongolia1, Mongolia2)		2		1960
	Turkey (NH insan, Beygir CAU, Uludag)		6		1970–1984
	India (NCTC 3708, NCTC 3709)		2		1931–1932
	China (NCTC 10245, ATCC 23344, EY2233, EY2235, EY2236, EY2237, EY2238 and EY2239)		8		1942–1951
	Hungary (NCTC 10229, NCTC 10230)		2		1970–1984
	Unknown (EY100)		1		Unknown
*B*. *thailandensis* (N = 4)	Thailand (E175, E264, E421, E426)			4	1991–2001
*B*. *thailandensis* variants with *B*. *pseudomallei-* like capsular polysaccharide (N = 6)	Thailand (SBXCC001, SBXCC005a, SBXCB001, SBXPL007a, SBXPR001)			5	2014–2015
	Cambodia (E555)			1	2005
*B*. *cepacia* (N = 8)	Thailand (U668, 10223, NCTC 10744, MI1035)	4			Unknown
	Thailand (2.1B, SBCAU015)			2	Unknown
	Lao PDR (39628)	1			Unknown
	Lao PDR (LNT40)			1	Unknown
*B*. *humptydooensis* (N = 1)	Australia (MSMB43)	1			Unknown
*B*. *oklahomensis* (N = 3)	Oklahoma, USA (c6786)	1			1973
	Oklahoma, USA (c7532, c7533)			2	1973
*B*. *multivorans* (N = 1)	UK (LMG16660)	1			Unknown
*B*. *vietnamiensis* (N = 1)	Vietnam (LMG6999)	1			Unknown
*B*. *ubonensis* (N = 1)	Thailand (DMST866)			1	Unknown

### Latex agglutination test and multilocus sequence typing

Latex agglutination reagent based on the 4B11 monoclonal antibody to capsular polysaccharide of *B*. *pseudomallei* was prepared and the assay performed as previously described [11, 35]. Multilocus sequence type (ST) was known for 21 *B*. *pseudomallei*, 21 *B*. *mallei* and 10 *B*. *thailandensis* isolates, which have been reported previously [13, 36, 37].

### MALDI-TOF MS analysis

Protein was extracted from bacteria using the formic acid extraction method as previously described [38, 39], with several modifications. In brief, a loopful of bacteria was harvested from an agar plate and suspended in 300 μl ultra-pure distilled water (UDW), to which 900 μl absolute ethanol (Merck, Darmstadt, Germany) was added. The suspension was centrifuged at 16,200 g for 2 min and the pellet left to dry at room temperature. Twenty-five microliters of 70% formic acid (V/V) (Sigma-Aldrich, Fluka, MO, USA) was added and mixed thoroughly, followed by an equal volume of acetonitrile (Merck, Darmstadt, Germany). The mixture was centrifuged at 16,200 g for 2 min, and then 1 μl of supernatant was spotted onto a MSP-384 polished steel target plate (Bruker Daltonics, Germany). After drying in air, all spots were overlaid with 1 μl of matrix, α-cyano-4-hydroxycinnamic acid (HCCA) (Bruker Daltonics, Germany) dissolved in a solution of 50% acetonitrile, 2.5% trifluoroacetic acid and 47.5% water (Sigma-Aldrich, Fluka, MO, USA). Each spot was measured in 200 shot steps for a total of 1200 laser shots using an MALDI-TOF Mass Spectrometer Autoflex speed (Bruker Daltonics, Germany) and FlexControl software (version 3.4.135, Bruker Daltonics, Germany). Spectra were obtained in the linear positive mode with an accelerating voltage of 19.5 kV and analyzed within a mass range of 2,000–20,000 Da. Before measurement, the instrument was calibrated using the Bacterial Test Standard (BTS) following the manufacturer’s instructions (Bruker Daltonics, Germany). *E*. *coli* DH5-Alpha was used as a positive control and matrix solution was used as a negative control. Identification was achieved using the MALDI-Biotyper software (version 3.1, Bruker Daltonics, Germany). These experiments were performed in duplicate and the scores were averaged. Interpretation was performed according to the manufacturer’s recommendation; score of ≥ 2.3, reliable species level identification; 2.0–2.29, probable species level identification; 1.7–1.9, probable genus level identification; ≤ 1.7, unreliable identification [40]. In a pilot study, at least 4 colonies (size ~ 1 mm) were required for *B*. *pseudomallei* to be identified with a score of ≥ 2.3.

### MALDI-TOF MS reference database

The Bruker Daltonics database (version 3.3.1.0) was expanded by adding main spectrum profiles (MSP) for 26 isolates after adjusting the baseline and smoothness using Flexanalysis software (version 3.4, Bruker Daltonics, Germany). Twenty spots of a single protein extract from each isolate were used to construct the MSP using the MALDI-Biotyper software (version 3.1). The isolates used were as follows: *B*. *pseudomallei* (K96243, H2660a, H2708a, 1106a and 1026b), *B*. *cepacia* (LNT40, 10223, 2.1B, 39628, MI1035, NCTC 10744, SBCAU015 and U668), *B*. *humptydooensis* (MSMB43), *B*. *mallei* (NCTC 10247 and Mongolia 1), *B*. *multivorans* (LMG16660), *B*. *oklahomensis* (c6786, c7532 and c7533), *B*. *thailandensis* (E175, E264, E421 and E426), *B*. *ubonensis* (DMST866) and *B*. *vietnamiensis* (LMG6999). The five *B*. *pseudomallei* isolates were selected as these have been used extensively as reference isolates, and whole genome sequence and sequence type (ST) data are available (K96243, ST10; H2660a, ST 54; H2708a, ST60; 1106a, ST70; and 1026b, ST102). These STs also represent the major *B*. *pseudomallei* STs in Thailand [37]. *B*. *mallei* evolved from a single lineage of *B*. *pseudomallei* and the two isolates selected here (NCTC 10247 and Mongolia 1) were used previously as reference isolates is a study that described the discrimination between *B*. *mallei* and *B*. *pseudomallei* [25]. *B*. *ubonensis*, *B*. *oklahomensis*, *B*. *humptydooensis* and *B*. *vietnamiensis* are rarely described and all isolates belonging to these species in our collection were used. *B*. *cepacia*, *B*. *multivorans* and *B*. *thailandensis* were added since they may be misidentified as *B*. *pseudomallei* by some identification systems.

### Identification of specific peaks for the discrimination of *Burkholderia* species

The Clinprotools software (version 3, Bruker Daltonics, Germany) was used to identify peaks that discriminated between *Burkholderia* species. This was performed using 20 replicates of a single protein preparation from each of nine *Burkholderia* species and *B*. *thailandensis* with Bp-like CPS: *B*. *multivorans* LMG16660, *B*. *ubonensis* DMST866, *B*. *vietnamiensis* LMG6999, *B*. *cepacia* NCTC 10744, *B*. *oklahomensis* c7533, *B*. *thailandensis* E264, *B*. *thailandensis* (Bp-like CPS) E555, *B*. *humptydooensis* MSMB43, *B*. *pseudomallei* K96243, and *B*. *mallei* NCTC 3708. Three independent experiments (20 replicates for each experiment) were performed and the results of each experiment checked for consistency of the discriminating peaks.

The statistical algorithms incorporated in the Clinprotools software (Quick Classifier (QC)/ Different Average, Supervised Neural Network (SNN) and the Genetic Algorithm (GA)) were used to analyze protein peaks between 2,000 and 20,000 Da. Potentially discriminatory peaks were identified based on high reliable prediction and separation based on cross validation values (>90%), as described previously [30]. Three statistical tests (Anderson-Darling (AD), t-test/ANOVA (TTA), and Wilcoxon/Kruskal-Wallis (W/KW) are incorporated into Clinprotools and were used to analyse intensity data. All discriminatory peaks that exhibited P-values of <0.01 by any of these statistical test were further evaluated by eye [41] and validated with the peaks of other isolates from the same species. Peaks that were unique to specific species or statistically different in intensity and showed consistent results from three independent experiments were considered as the discriminatory peaks. All *Burkholderia* isolates used the for identification of discriminating peaks were used for the construction of a dendrogram.

### MALDI-TOF MS dendrogram

**Fig 1:**
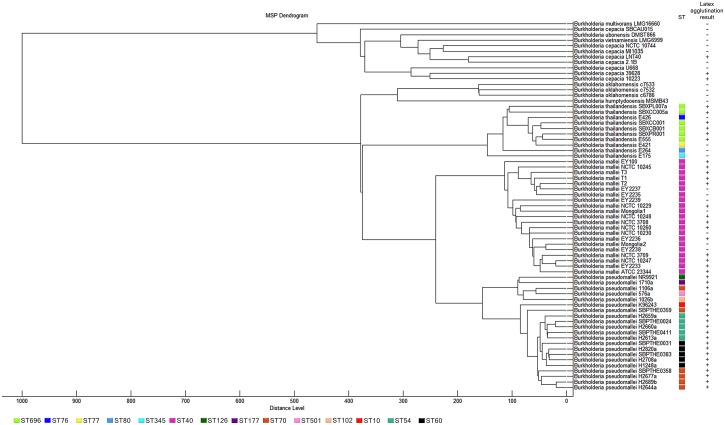
Phylogenetic tree of protein profiles of *B*. *pseudomallei* and 8 other genetically related *Burkholderia* species. Distance is displayed in relative units. Annotated with MLST sequence type (ST) where known; each color represents a ST. The latex agglutination test was based on a monoclonal that recognizes *B*. *pseudomallei* capsular polysaccharide.

To construct the dendrogram, Flexanalysis software (version 3.4) was used to adjust the baseline and smoothness of the spectra. Twenty spots of a single protein extract for each isolate were used to construct the main spectrum profiles (MSP) using the MALDI-Biotyper software (version 3.1). The following parameters were used: the Biotyper MSP creation standard method was used, with a maximum mass error of each single spectrum of 2000, desired mass error for the MSP of 200, desired peak frequency minimum of 25%, and maximum desired peak number for the MSP of 70. The isolates used were: 21 *B*. *pseudomallei* (NR9921, 1710a, 1106a, 576a, 1026b, K96243, SBPTHE0359, H2659A, SBPTHE0024, H2660a, SBPTHE0411, H2613a, SBPTHE0031, H2820a, SBPTHE0383, H2708a, H1248a, SBPTHE0358, H2677a, H2689b, H2644a), 21 *B*. *mallei* (EY100, NCTC 10245, T1, T2, T3, EY2233, EY2235, EY2236, EY2237, EY2238, EY2239, NCTC 10229, NCTC 10248, NCTC 3708, NCTC 10230, NCTC 10260, NCTC 3709, NCTC 10247, Mongolia1, Mongolia 2, ATCC 23344), 10 *B*. *thailandensis* (E175, E264, E421, E426, E555, SBXPL007a, SBXCC006a, SBXCC001, SBXCB001, SBXPR001), 1 *B*. *humptydooensis* MSMB43, 3 *B*. *oklahomensis* (c6786, c7532, c7533), 8 *B*. *cepacia* (NCTC 10744, MI1035, LNT40, 2.1B, U668, 39628, 10223, SBCAU015), 1 *B*. *vietnamiensis* LMG6999, 1 *B*. *ubonensis* DMST866 and 1 *B*. *multivorans* LMG16660. The basis for the choice of *B*. *pseudomallei* isolates was the inclusion of commonly used reference isolates (K96243, H2660a, H2708a, 1106a and 1026b) and a further 16 isolates each assigned to a different ST (ST696, ST76, ST77, ST80, ST345, ST40, ST126, ST177, ST70, ST501, ST102, ST10, ST54 and ST60 (Fig 1). *B*. *thailandensis* and *B*. *thailandensis* with BP-like CPS capsule were randomly selected from our freezer archive. We included all of the *B*. *mallei* (N = 21), *B*. *humptydooensis* MSMB43 (N = 1), *B*. *oklahomensis* (N = 3), *B*. *cepacia* (N = 8), *B*. *vietnamiensis* (N = 1), *B*. *ubonensis* (N = 1) and *B*. *multivorans* (N = 1) in our collection. Cluster analysis was performed based on comparison of MSP using the following setting parameters: the distance measure was set to Spearman, the linkage was set to single, and score threshold value for a single organism was set at 1000.

### Determination of minimum bacterial input for accuracy of MALDI-TOF

The minimum number of bacteria in suspension and minimum number of colonies required to achieve an accurate MALDI-TOF result were determined. To quantify the number of bacteria, the experiment was performed using bacterial suspension. *B*. *pseudomallei* K96243 was harvested from an overnight culture of Columbia agar, suspended in sterile saline, adjusted to approximately 1 x 10^7^ CFU/ml and then serially diluted from 1 x 10^7^ CFU/ml to 10 CFU/ml. Bacterial cells in 1ml of each dilution were harvested by centrifugation at 16,200 g for 2 min. Protein was extracted from each pellet prior to MALDI-TOF analysis as above. The bacterial count was confirmed using Columbia agar spread plates in triplicate and colony counts after overnight incubation at 37°C. To determine the minimum number of colonies required for MALDI-TOF, between 1 and 10 colonies (size ~1 mm) of *B*. *pseudomallei* K96243 were harvested from Columbia agar plates using a loop, suspended in UDW and the protein extracted from each. Extracts were analyzed in triplicate in both assays. Spectra with maximal absolute peak intensities ranging from 10^3^ to 10^4^ arbitrary units were considered for evaluation [42, 43].

### Effect of culture media

The effect of culture media was examined for five *B*. *pseudomallei* isolates (K96243, NR9921, 1106a, 1026b and 576a). Bacteria were streaked onto Ashdown agar, blood agar, Columbia agar, chocolate agar, Luria-Bertani (LB) agar, MacConkey agar, Mueller-Hinton agar, trypticase soy agar and incubated at 37°C in air for 24 hours and harvested using a 1 μl loop (≥ 10 colonies, size ~1 mm). Bacteria were suspended in 300 μl UDW and the protein extracted and analyzed in triplicate using MALDI-TOF MS.

### MALDI-TOF MS identification of *B*. *pseudomallei* in spiked blood culture

Three BACTEC Plus Aerobic/F bottles were each inoculated with 10 ml of whole blood drawn from a single healthy volunteer. An overnight culture of *B*. *pseudomallei* K96243 was adjusted to approximately 1 x 10^8^ CFU/ml, serially diluted to a concentration of 100 CFU/ml, and 100 μl (10 CFU) inoculated into each bottle. The inoculum was confirmed using colony counts on agar plates. Bottles were incubated at 37°C with 200 rpm shaking. At 12, 16, 20 and 24 h after incubation, one ml from each bottle was withdrawn for protein extraction using ammonium 0.826% NH_4_Cl (W/V) as a lysis buffer, as described previously [44, 45] with modifications. Briefly, an equal volume of 0.826% NH_4_Cl was added to 1 ml of blood culture fluid, mixed and centrifuged at 16,200 g for 2 min. The pellet was lysed twice as above before washing twice with 1 ml ultrapure distilled water and centrifuged as before. The supernatant was discarded and the pellet used for protein extraction.

### *RecA* sequencing

*recA* sequencing and phylogenetic analysis was performed as previously described [20]. The isolates tested were *B*. *pseudomallei* (N = 6), *B*. *mallei* (N = 7), *B*. *thailandensis* (N = 2), *B*. *thailandensis* variant strains with Bp-like CPS (N = 1), *B*. *ubonensis* (N = 1), *B*. *oklahomensis* (N = 1), *B*. *vietnamiensis* (N = 1), *B*. *cepacia* (N = 5), *B*. *humptydooensis* (N = 1) and *B*. *multivorans* (N = 1). Genomic DNA was extracted and PCR amplification performed using BUR3, BUR4 and BUR5 primers as previously described [20]. Products were visualized using agarose-gel electrophoresis and purified using ExoSAP-IT PCR Product Cleanup (Affymetrix UK Ltd., UK). Purified PCR products were sequenced by Macrogen Inc. (Korea). Nucleotide sequence of *B*. *thailandensis* E555 was obtained from the NCBI database (accession no. AECN01000010.1). All sequences were aligned and trimmed to a 348 bp region using Clustal W using MEGA software version 7 [46]. A maximum likelihood tree was constructed using the Nearest-Neighbor-Interchange (NNI) and Tamura-Nei model [47] using MEGA software version 7.0.14 [46].

### Ethical approval

The study was approved by Ethics Committee of the Faculty of Tropical Medicine, Mahidol University (approval number MUTM 2016-034-01). The principal investigator's blood was used and verbal consent was obtained to participate in this study. The Ethics Committee of the Faculty of Tropical Medicine, Mahidol University approved the procedure.

## Results

### Evaluation of MALDI-TOF MS for the identification of *B*. *pseudomallei*

Twenty replicates of *B*. *pseudomallei* K96243 were tested using MALDI-TOF MS and the Bruker Daltonics database version 3.3.1.0. These were all identified as *B*. *thailandensis*, with a median score below that for reliable species identification (median 1.96, range, 1.83–2.04). We noted that the Biotyper database did not contain *B*. *pseudomallei*, *B*. *mallei*, *B*. *ubonensis*, *B*. *oklahomensis* or *B*. *humptydooensis*, although did contain representation for 30 *Burkholderia* species (*B*. *ambifaria* (N = 2), *B*. *andropogonis* (N = 1), *B*. *anthin*a (N = 2), *B*. *caledonica* (N = 1), *B*. *caribensis* (N = 1), *B*. *cenocepacia* (N = 2), *B*. *cepacia* (N = 9), *B*. *diffusa* (N = 1), *B*. *dolosa* (N = 1), *B*. *fungorum* (N = 1), *B*. *gladioli* (N = 5), *B*. *glathei* (N = 1), *B*. *glumae* (N = 1), *B*. *lata* (N = 1), *B*. *latens* (N = 1), *B*. *metallica* (N = 1), *B*. *multivorans* (N = 5), *B*. *phenazinium* (N = 1), *B*. *phymatum* (N = 1), *B*. *plantarii* (N = 1), *B*. *pyrrocinia* (N = 2), *B*. *sacchari* (N = 1), *B*. *seminalis* (N = 2), *B*. *stabilis* (N = 2), *B*. *terricola* (N = 1), *B*. *tropica* (N = 1), *B*. *tuberum* (N = 1), *B*. *thailandensis* (N = 1), *B*. *vietnamiensis* (N = 1) and *B*. *xenovorans* (N = 1)). We extended the database by adding reference profiles for *B*. *pseudomallei* (N = 5), *B*. *mallei* (N = 2), *B*. *ubonensis* (N = 1) *B*. *oklahomensis* (N = 3) and *B*. *humptydooensis* (N = 1), together with further examples of *B*. *cepacia* (N = 8), *B*. *thailandensis* (N = 4), *B*. *multivorans* (N = 1), and *B*. *vietnamiensis* (N = 1). We then tested the accuracy of MALDI-TOF MS for the identification of geographically and genetically diverse *B*. *pseudomallei* isolates. A large collection of 564 clinical and 17 environmental isolates from different locations in Thailand, and from Laos, Cambodia, Hong Kong and Australia was tested. MALDI-TOF identified all 581 *B*. *pseudomallei* isolates correctly (100% sensitivity), with a median score for all isolates of 2.49 (range 2.30–2.68, IQR, 2.43–2.54) and no misidentification at the species level (Table 2). *B*. *pseudomallei* isolates showed some variability in similarity scores against the 5 *B*. *pseudomallei* added here to the reference profiles. Despite this, the highest score was to *B*. *pseudomallei* for all 581 *B*. *pseudomallei* tested, with the second and third highest score being *B*. *mallei* (median score 2.21, range 1.68–2.46, IQR 2.14–2.29) and *B*. *thailandensis* (median score 2.01, range 1.62–2.22, IQR 1.95–2.06). The specificity of MALDI-TOF MS was evaluated by testing 25 isolates including *B*. *mallei* (N = 19), *B*. *thailandensis* (N = 6), and 23 isolates belonging to 21 species in other Genera (see Methods). These bacterial species were correctly identified, indicating 100% specificity.

**Table 2:** MALDI-TOF scores of *B*. *pseudomallei* from different geographical origins.

Locations		No. of isolates tested	Range	Median	IQR
Northeast Thailand	Ubon Rachathani	443	2.30–2.68	2.51	2.45–2.55
	Udon Thani	35	2.32–2.52	2.43	2.38–2.46
	Khon Kaen	19	2.34–2.50	2.40	2.37–2.44
	Nakhon Phanom	19	2.33–2.53	2.44	2.38–2.48
East Thailand	Chachoengsao	8	2.34–2.49	2.39	2.35–2.42
Laos		12	2.46–2.66	2.52	2.48–2.60
Cambodia		14	2.35–2.57	2.47	2.44–2.50
Hong Kong		4	2.56–2.58	2.56	2.56–2.57
Australia		27	2.32–2.58	2.42	2.38–2.44
All isolates		581	2.30–2.68	2.49	2.43–2.54

### Effect of culture media on MALDI-TOF MS identification

We evaluated whether the culture medium used to grow bacteria prior to MALDI-TOF MS affected the accuracy of identification. Five *B*. *pseudomallei* strains (NR9921, 1106a, 576a, K96243, 1026b) were grown overnight on eight different solid media. All isolate/media combinations were identified as *B*. *pseudomallei*, with an identification score of at least 2.45. The median scores (range) of five isolates were as follows: Ashdown agar, 2.59 (2.48–2.68); blood agar, 2.66 (2.49–2.75); Columbia agar, 2.65 (2.59–2.69); chocolate agar, 2.64 (2.48–2.67); Luria-Bertani (LB) agar, 2.65 (2.47–2.70); MacConkey agar, 2.59 (2.47–2.62); Mueller-Hinton agar, 2.63 (2.45–2.65); and trypticase soy agar 2.62 (2.49–2.67).

### Identification of *B*. *pseudomallei* in spiked blood culture

Since the speed of identification of *B*. *pseudomallei* from blood culture is crucial for patient management, we tested whether MALDI-TOF MS would give an accurate identification of *B*. *pseudomallei* K96243 in spiked blood culture fluid. For this we used three 30 ml BACTEC Plus Aerobic/F blood culture bottles each containing 10ml of blood from a healthy volunteer, spiked with 10 CFU, and tested after 8, 10, 12 and 24 h of incubation at 37°C with shaking. Testing at 8, 10, 12 h showed unidentified or misidentified results, but accurate identification for *B*. *pseudomallei* was obtained at the 24 h time point (median identification score 2.25 (range 2.17–2.36)). Colony counting demonstrated that the bacterial concentration in the blood culture bottle fluid at 24 h was 2.1 x 10^9^ CFU/ml.

### Latex agglutination test

The latex agglutination test is used to rapidly identify *B*. *pseudomallei* colonies in our laboratory and elsewhere, and so we performed a comparative assessment of this versus MALDI-TOF MS. The isolates used were 21 *B*. *pseudomallei*, 21 *B*. *mallei*, 10 *B*. *thailandensis*, 3 *B*. *oklahomensis*, 8 *B*. *cepacia*, 1 *B*. *vietnamiensis*, 1 *B*. *ubonensis* and 1 *B*. *multivorans*. All *B*. *pseudomallei* were positive by latex agglutination (Fig 1), as well as all *B*. *thailandensis* variants expressing Bp-like CPS, some strains of *B*. *mallei* (14/21, 66.7%), and three strains of *B*. *cepacia* (LNT40, 39628 and 10223) which had previously given false-positive results (D. Dance, personal communication).

### Identification of discriminatory peaks

**Fig 2:**
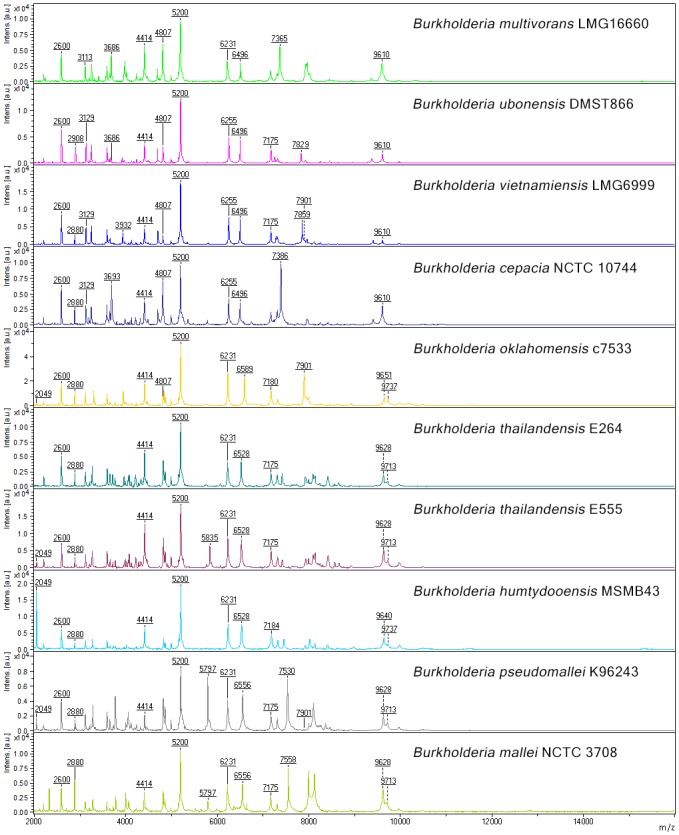
MALDI-TOF mass protein spectra of *B*. *pseudomallei* and 8 other genetically related *Burkholderia* species. The vertical axis shows relative intensities of ions and the horizontal axis shows mass to charge ratio (m/z) or masses of ions (Da).

Twenty discriminatory peaks were identified from MSP spectra of representative isolates from 9 *Burkholderia* species and *B*. *thailandensis* with Bp-like CPS with cross validation results ranging between 98% and 100% (Fig 2 and S1 Table). Three peaks (around 2,600 Da, 4,414 Da and 5,200 Da) were observed for all nine *Burkholderia* species. Peaks that were unique to specific species were also observed, for example 2,908 Da for *B*. *ubonensis*, 3,932 Da for *B*. *vietnamiensis*, and 5,835 Da for *B*. *thailandensis* with Bp-like CPS. The only difference between *B*. *thailandensis* and *B*. *thailandensis* with Bp-like CPS was a peak of 5,835 Da. Compared to other *Burkholderia* species, peaks with highest intensity that were statistically different (P < 0.001) and useful to differentiate species were 2,049 Da for *B*. *humptydooensis*, 5,797 for *B*. *pseudomallei*, 7,558 Da for *B*. *mallei* and 7,859 Da for *B*. *vietnamiensis*. We checked these peaks with other isolates [*B*. *pseudomallei* (N = 21), *B*. *mallei* (N = 21), *B*. *thailandensis* (N = 4), *B*. *thailandensis* with Bp-like CPS (N = 6), *B*. *oklahomensis* (N = 3), *B*. *cepacia* (N = 8)] and found them to be reproducible in other isolates of the same species.

### Phylogeny based on MALDI-TOF MS protein profiles and *recA*

A phylogenetic tree based on the MSP of 67 *Burkholderia* isolates divided nine species into two major branches (Fig 1). The first contained *B*. *multivorans*, *B*. *vietnamiensis*, *B*. *ubonensis* and *B*. *cepacia*, and the second contained *B*. *thailandensis*, *B*. *oklahomensis*, *B*. *humptydooensis*, *B*. *mallei* and *B*. *pseudomallei*. *B*. *pseudomallei* and *B*. *mallei* resided in distinct lineages but were more related to each other than to *B*. *thailandensis*. Sequence types had been defined previously for the *B*. *mallei* and *B*. *pseudomallei* isolates [36, 37] and are shown in Fig 1. *B*. *mallei* isolates belonged to a single ST (ST40), while *B*. *pseudomallei* belonged to 8 different STs.

**Fig 3:**
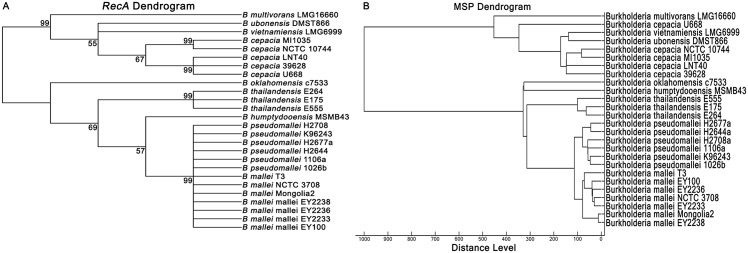
Comparison of *recA* sequence dendrogram (A) and protein profile dendrogram (B) of *B*. *pseudomallei* and 8 other genetically related *Burkholderia* species.

A phylogenetic tree based on *recA* sequence was compared with the MALDI-TOF dendrogram for 26 isolates representing nine *Burkholderia* species (Fig 3). This demonstrated a broadly similar structure between the two, with distribution of the species between two major divisions. A notable difference was that MS distinguished between *B*. *pseudomallei* and *B*. *mallei*, while the *recA* tree did not.

## Discussion

MALDI-TOF MS is increasingly used for bacterial identification in diagnostic microbiology laboratories. This technology can reduce time of identification since bacteria are sampled directly from bacterial colonies and the test takes around 30 minutes to perform. The rapid microbiological diagnosis of melioidosis is essential because clinical features are non-specific, empiric drug regimens are sub-optimal, and infection is often fatal without appropriate treatment. This is compounded by the fact that healthcare providers in non-endemic countries may not readily recognize melioidosis. MALDI-TOF MS could also be used for environmental surveys looking at the distribution and presence of *B*. *pseudomallei* and related species.

Our findings confirm that MALDI-TOF MS can accurately identify *B*. *pseudomallei* regardless of geographic origin, and was able to reliably distinguish between this, other *Burkholderia* spp. and a range of other common pathogens, provided that the database is suitably modified. Since the top hit identification for each tested *B*. *pseudomallei* isolates could be any of five *B*. *pseudomallei* isolates in our reference database, we recommended laboratories that wish to identify *B*. *pseudomallei* using this method to extend the database by adding reference profiles of all five *B*. *pseudomallei* isolates. Diagnostic laboratories use a range of culture media, but we demonstrated that this has no effect on the performance of the test. We also showed that MALDI-TOF MS accurately identifies *B*. *pseudomallei* in blood cultures, which could reduce the time taken to diagnostic confirmation and appropriate antimicrobial treatment. Our results confirm a previous report in which MALDI-TOF was used to identify *B*. *pseudomallei* from blood cultures from two septicemic patients in Australia [29]. The simulated blood culture experiment in which 10 CFU was used as the starting inoculum reflects the bacterial load in blood during human infection, which has been reported previously to be a median count of 1.1 CFU/ml [48]. Our observation that identification of *B*. *pseudomallei* by MALDI-TOF was not accurate until after an incubation period of 24 hours implies that in clinical practice, this method will be effective on bottles that have flagged in an automated incubation system or after 24 hours or more of incubation.

The Clinprotools software used to support the interpretation of MALDI-TOF MS identifies species-specific peaks as the basis for species identification [30, 41, 49]. In agreement with previous studies [25, 30], we identified specific peaks at 4,410, 5,794, 6,551, 7,553 and 9,713 Da for all *B*. *pseudomallei* isolates tested. We also observed specific peaks for *B*. *ubonensis* (2,908 Da), *B*. *vietnamiensis* (3,932 Da) and *B*. *thailandensis* variants expressing Bp-like CPS (5,835 Da). The peak at 2,049 Da may be used to differentiate between *B*. *pseudomallei* and *B*. *mallei* because this was present in all *B*. *pseudomallei* isolates but not in any *B*. *mallei* isolates. We also noted several peaks which displayed significantly higher peak intensities in specific species, which may be useful for the discrimination of *B*. *humptydooensis* (2,049 Da), *B*. *multivorans* (3,686 Da), *B*. *pseudomallei* (5,797 Da), *B*. *oklahomensis* (6,589 and 7,901 Da) and *B*. *vietnamiensis* (7,859 Da).

Latex agglutination is a sensitive screening test for suspected *B*. *pseudomallei*, but positive latex agglutination results have been described previously for *B*. *mallei*, *B*. *thailandensis* with Bp-like CPS, *S*. *aureus* [11, 35, 50] and some strains of *B*. *cepacia* (unpublished data), potentially leading to confusion in diagnostic laboratories if used alone. This study shows that MALDI-TOF MS can be used to reliably distinguish between these organisms. Furthermore, both MSP and *recA* dendrograms confirmed that *B*. *pseudomallei*, *B*. *mallei* and *B*. *thailandensis* were arranged in the same phylogenetic group.

In conclusion, MALDI-TOF MS is an accurate and discriminatory tool for the identification of *B*. *pseudomallei* if sufficient MSPs are added in the Biotyper database. MALDI-TOF MS could be used to increase the rapid detection of cases of melioidosis in the clinical setting, and reduce time to appropriate antimicrobial therapy.

## Supporting information

S1 Table Differentiating peaks of nine *Burkholderia* species. Peaks were based on analysis using Clinprotools software.
(PDF)
