# Supplementary material for: Matrix-assisted laser desorption/ionization time-of-flight mass spectrometry for the identification of Burkholderia pseudomallei from Asia and Australia and differentiation between Burkholderia species
Source: PLoS One. 2017 Apr 6;12(4):e0175294. doi: 10.1371/journal.pone.0175294 (PMC5383291; doi:10.1371/journal.pone.0175294)
Supplement: S1 Table — Peaks were based on analysis using Clinprotools software. (PDF) [file pone.0175294.s001.pdf]

S1 Table Differentiating peaks of nine *Burkholderia* species. Peaks were based on analysis using Clinprotools software

	Peak mass (m/z) representing the protein size in Dalton (Da)			Mean of peak intensity ± standard variation (number of isolates positive).[a]									
No.	Mass	Start Mass	End Mass	*B. pseudomallei* (n = 21)	*B. mallei* (n = 21)	*B. humptydooensis* (n = 1)	*B. thailandensis* (n = 4)	*B. thailandensis* with Bp-like CPS (n = 6)	*B. oklahomensis* (n = 3)	*B. cepacia* (n = 8)	*B. vietnamiensis* (n = 1)	*B. ubonensis* (n = 1)	*B. multivorans* (n = 1)
1	2,049.05	2,043.54	2,061.63	4.5 ± 3.2 (21)	–	**45.3 ± 6.1** (1)	2.2 ± 0.7 (3)	4.9 ± 2.9 (5)	2.8 ± 0.5 (3)	–	–	–	–
2	2,600.11	2,586.53	2,620.05	**15.3 ± 5.9** (21)	**15.4 ± 5.9** (21)	**19.8 ± 3.6** (1)	**19.5 ± 5.7** (4)	**18.6 ± 3.6** (6)	**19.6 ± 4.7** (3)	**21.6 ± 5** (8)	**37.6 ± 4.6** (1)	**37.1 ± 5.3** (1)	**44.3 ± 7.9** (1)
3	2,880.26	2,870.93	2,890.21	7 ± 2.2 (21)	**20.9 ± 8.5** (21)	6.7 ± 0.7 (1)	9.7 ± 1.5 (4)	8.1 ± 1.7 (6)	**12.2 ± 3.3** (3)	7.9 ± 3 (8)	8.9 ± 1.1 (1)	–	–
4	2,908.54	2,900.32	2,915.76	–	–	–	–	–	–	–	–	**20.4 ± 2.7** (1)	–
5	3,129.48	3,123.16	3,152.95	–	–	–	–	–	–	**14.3 ± 4.2** (8)	**21.2 ± 2.8** (1)	**24.7 ± 2.8** (1)	–
6	3,686.95	3,678.28	3,691.38	–	–	–	–	–	–	–	–	2.8 ± 0.2 (1)	**52.9 ± 9.2** (1)
7	3,932.56	3,922.99	3,940.71	–	–	–	–	–	–	–	**15.1 ± 2.6** (1)	–	–
8	4,414.72	4,388.77	4,426.66	**19 ± 4.1** (21)	**18.3 ± 8.1** (21)	**18.2 ± 1.2** (1)	**29.8 ± 8.7** (4)	**30.9 ± 6.6** (6)	**18.2 ± 3.7** (3)	**17.3 ± 5.1** (8)	**22.9 ± 2.9** (1)	**23.7 ± 1.9** (1)	**18.8 ± 2.3** (1)
9	4,807.06	4,787.26	4,811.52	–	–	–	–	–	6.6 ± 1.3 (3)	**21.8 ± 5.4** (8)	**14.7 ± 1.7** (1)	**22.7 ± 2** (1)	**24 ± 4.6** (1)
10	5,200.32	5,187.87	5,213.46	**33.5 ± 11.6** (21)	**34.5 ± 9.1** (21)	**57.6 ± 4** (1)	**53.6 ± 13.2** (4)	**52.9 ± 7.4** (6)	**54.2 ± 7.2** (3)	**40.9 ± 7.8** (8)	**96.3 ± 9.3** (1)	**84 ± 4.2** (1)	**53.4 ± 7.2** (1)
11	5,797.16	5,776.06	5,811.11	**38.6 ± 14.4** (21)	3.3 ± 1.7 (21)	–	–	–	–	2.2 ± 0.8 (8)	2.5 ± 0.4 (1)	2.1 ± 0.3 (1)	2.1 ± 0.4 (1)
12	5,835.21	5,820.98	5,851.76	–	–	–	–	**15.2 ± 8.9** (6)	–	–	–	-	–
13	6,231.61	6,193.04	6,244.07	**15.3 ± 4.8** (21)	**14.4 ± 3.9** (21)	**25.3 ± 1.9** (1)	**25 ± 7.1** (4)	**24.6 ± 3.2** (6)	**25.4 ± 4.8** (3)	–	–	-	**18.7 ± 2.2** (1)
14	6,496.03	6,459.94	6,506.02	3.7 ± 0.8 (21)	3.1 ± 0.7 (21)	6.6 ± 0.3 (1)	5.8 ± 1.1 (4)	6.1 ± 0.7 (6)	–	**17.1 ± 5.7** (8)	**38.2 ± 4.2** (1)	**34.1 ± 2.6** (1)	**15.4 ± 2.1** (1)
15	6,528.31	6,517.25	6,546.71	8.6 ± 1.7 (21)	5.4 ± 0.9 (4)	**23.8 ± 1.6** (1)	**23.8 ± 6.5** (4)	**23.8 ± 3** (6)	–	–	–	–	–
16	6,556.5	6,546.71	6,580.51	**16.1 ± 4.4** (21)	**17.2 ± 3.9** (21)	–	–	–	–	–	–	–	–
17	6,589.47	6,580.51	6,631.97	6.6 ± 0.9 (3)	5.8 ± 0.7 (12)	–	–	–	**22.2 ± 4** (3)	–	–	–	–
18	7,558.59	7,545.03	7,618.52	**17.4 ± 9.1** (21)	**32.1 ± 17.4** (21)	–	–	–	–	–	–	–	–
19	7,859.45	7,840.27	7,875.56	–	–	–	–	–	–	–	**32.6 ± 2.3** (1)	4.6 ± 0.5 (1)	–
20	7,901.15	7,875.56	7,913.91	4.6 ± 1.7 (5)	–	–	–	–	**27.2 ± 3.7** (3)	–	8.9 ± 0.5 (1)	–	–

[a] peak present; –, no peak observed
Bold font indicate intensity > 10
